# Improved Isolation of Mesenchymal Stem Cells Based on Interactions between *N*-Acetylglucosamine-Bearing Polymers and Cell-Surface Vimentin

**DOI:** 10.1155/2019/4341286

**Published:** 2019-11-11

**Authors:** Hirohiko Ise, Kumiko Matsunaga, Marie Shinohara, Yasuyuki Sakai

**Affiliations:** ^1^Institute for Materials Chemistry and Engineering, Kyushu University, CE41-206, 744 Motooka Nishi-ku, Fukuoka 819-0395, Japan; ^2^Somar Corp., 4-11-2 Ginza Chuo-ku, Tokyo 104-8109, Japan; ^3^Institute of Industrial Science, The University of Tokyo, Fe505, 4-6-1 Komaba Meguro-ku, Tokyo 153-8505, Japan

## Abstract

Mesenchymal stem cells (MSCs) in bone marrow and adipose tissues are expected to be effective tools for regenerative medicine to treat various diseases. To obtain MSCs that possess both high differentiation and tissue regenerative potential, it is necessary to establish an isolation system that does not require long-term culture. It has previously been reported that the cytoskeletal protein vimentin, expressed on the surfaces of multiple cell types, possesses *N*-acetylglucosamine- (GlcNAc-) binding activity. Therefore, we tried to exploit this interaction to efficiently isolate MSCs from rat bone marrow cells using GlcNAc-bearing polymer-coated dishes. Cells isolated by this method were identified as MSCs because they were CD34-, CD45-, and CD11b/c-negative and CD90-, CD29-, CD44-, CD54-, CD73-, and CD105-positive. Osteoblast, adipocyte, and chondrocyte differentiation was observed in these cells. In total, yields of rat MSCs were threefold to fourfold higher using GlcNAc-bearing polymer-coated dishes than yields using conventional tissue-culture dishes. Interestingly, MSCs isolated with GlcNAc-bearing polymer-coated dishes strongly expressed CD106, whereas those isolated with conventional tissue-culture dishes had low CD106 expression. Moreover, senescence-associated *β*-galactosidase activity in MSCs from GlcNAc-bearing polymer-coated dishes was lower than that in MSCs from tissue-culture dishes. These results establish an improved isolation method for high-quality MSCs.

## 1. Introduction

Mesenchymal stem cells (MSCs) have the potential to differentiate into osteocytes, chondrocytes, adipocytes, hepatocytes, nerve cells, and vascular endothelial cells [1–6]. Therefore, they are promising sources for regenerating cartilage and liver tissue [2–7]. Moreover, MSCs produce multiple effective cytokines, growth factors, and exosomes that promote tissue regeneration [8, 9]. However, these functions of MSCs tend to be unstable and difficult to maintain [10–13], likely as a result of long-term culture and high passage numbers. These problems create a need for isolation methods that do not require long-term culture. MSCs are relatively rare, accounting for only 0.001–0.01% of bone marrow and adipose tissues, the most abundant sources [14]. To obtain large numbers of MSCs without long-term culture, efficient isolation of MSCs with high proliferative activity is critical.

Most MSC isolation methods depend on nonspecific adhesion to tissue-culture dishes, with cultures subsequently established from these adherent cells [15, 16]. Therefore, to obtain sufficient MSCs for most therapeutic approaches, long-term culture is necessary. While this initial adhesion fractionation step is very simple and convenient, we hypothesized that affinity-based isolation methods would provide significant improvements. Recent studies have reported affinity-based isolation systems for MSCs using cell sorting and magnetic beads, exploiting MSC-specific markers, including CD73 and CD90 [17, 18]. However, these systems are expensive and have low yields. The purpose of this study was to develop a less-expensive, efficient, and convenient protocol for MSC isolation using selective adhesion. We focused on the cytoskeletal protein vimentin as an MSC marker.

Bone marrow is composed of hematopoietic, marrow adipose, and supportive stromal cells. Vimentin expression in bone marrow is specific to stromal cells [19], which include MSCs. Therefore, separation and enrichment of MSCs from bone marrow cells can be achieved by exploiting vimentin expression. Vimentin plays an important cytoplasmic role in maintaining cell architecture and positioning organelles. Although it has long been considered a cytosolic protein, cell-surface forms have recently been discovered on several cell types [20–25].

We have found that vimentin on the surfaces of several cell types possesses *N*-acetylglucosamine- (GlcNAc-) binding activity [26–29]. Moreover, multiple GlcNAc-bearing polymers interact with cancer cells, fibroblasts, and skeletal muscle cells via cell-surface vimentin [29, 30]. The latter half of the vimentin rod II domain has been identified as the GlcNAc-binding domain. It is expressed on the cell surface as tetrameric, but not filamentous, forms of vimentin [28]. We therefore hypothesized that efficient enrichment of MSCs from bone marrow cells could be achieved based on this interaction.

In this study, we first confirmed the expression of cell-surface vimentin on MSCs using an immortalized human line. Second, we prepared GlcNAc-bearing polymer-coated polystyrene dishes. Several GlcNAc-bearing polymers with different molecular weights were designed, and the coating conditions were optimized. Third, rat bone marrow cells were cultured on the optimal GlcNAc-bearing polymer-coated dishes, and colony formation and cell proliferation were quantitated. To confirm that proliferating cells had characteristics of MSCs, the expression of CD90, CD29, CD44, CD54, CD73, CD105, and CD106 (MSC-positive), as well as CD34, CD45, and CD11b/c (MSC-negative), markers were examined. Osteoblast, adipocyte, and chondrocyte differentiation was observed by Alizarin Red, Oil Red O, and Alcian Blue staining, respectively. We have established an efficient and convenient first step for the isolation of MSCs from bone marrow, which should have significant clinical utility.

## 2. Materials and Methods

### 2.1. Preparation of Polymers and Recombinant Vimentin for Coating Culture Dishes

**Figure 1:**
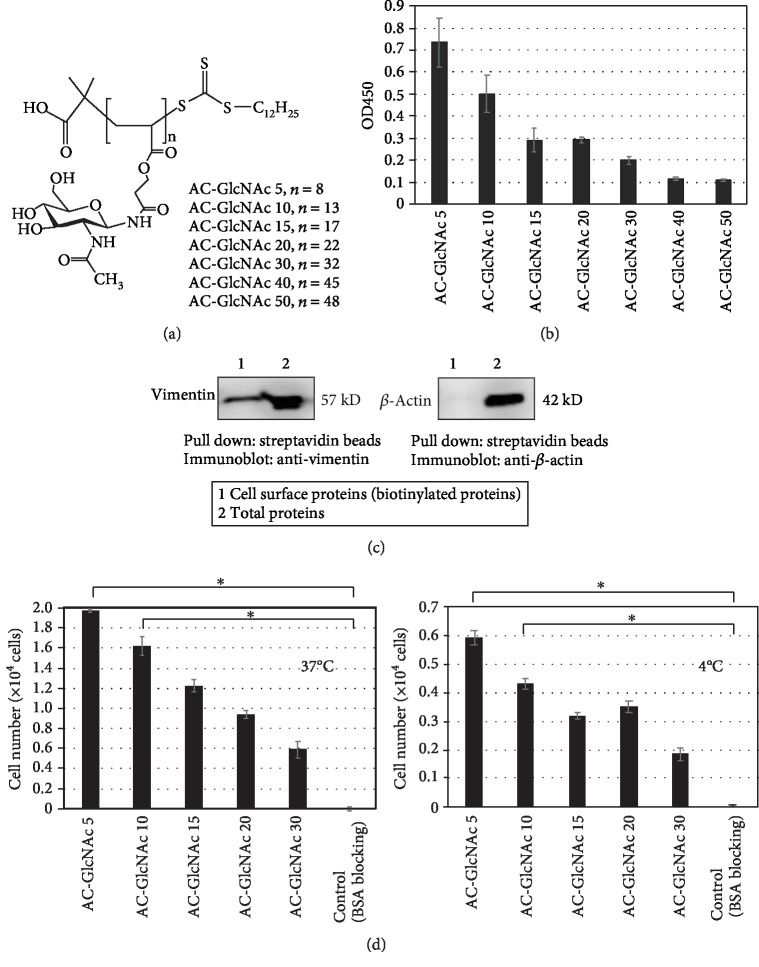
Evaluation of AC-GlcNAc coatings. (a) Structure of AC-GlcNAc. (b) Evaluation of AC-GlcNAc coating on polystyrene dishes using the interaction between biotinylated sWGA and AC-GlcNAc. The amount of biotinylated sWGA on dishes was measured by HRP-neutravidin and TMB solution (absorbance at 450 nm). (c) Western blots demonstrating expression of cell-surface vimentin in human immortalized MSCs. (d) UE7T-13 adherence to AC-GlcNAc-coated dishes at 37 (left) and 4°C (right). ^∗^*P* < 0.01, *n* = 3.

GlcNAc-bearing polymers (AC-GlcNAc) were synthesized according to previously described methods [29]. These polymers were composed of acrylate as the main chain and GlcNAc as the side chain (Figure 1(a)). To determine the optimal conditions for AC-GlcNAc coating on polystyrene dishes, AC-GlcNAc polymers of multiple sizes were prepared by polymerization with reversible addition-fragmentation chain transfer (RAFT) reagents. Briefly, AC-GlcNAc monomers were polymerized by 2,2′-azobis (isobutyronitrile) (AIBN; Tokyo Chemical Industry Co., Ltd., Tokyo, Japan) as an initiator and 2-(dodecylthiocarbonothioylthio)-2-methylpropanoic acid (DTMPA; Sigma, St. Louis, MO, USA) as the RAFT reagent. The molecular weight of AC-GlcNAc was controlled by altering the concentration of DTMPA. The molecular weights of AC-GlcNAc molecules were controlled by varying the molar ratio (5, 10, 15, 20, 30, 40, and 50) of AC-GlcNAc monomer to DTMPA, as measured by gel permeation chromatography (LC-9110G NEXT with the JAIGEL-GS510 Column; Japan Analytical Industry Co., Ltd., Tokyo, Japan) with 200 mM NaNO_3_/20% acetonitrile in water, and using a pullulan standard.

AC-GlcNAc-coated dishes were prepared by adding 50 *μ*L or 1 mL of 100 *μ*g/mL AC-GlcNAc to 96-well or 35-mm polystyrene dishes, respectively. The dishes were incubated with open lids overnight to dry the polymer solutions. In the case of rat bone marrow cell culture, to promote the proliferation of adherent cells, AC-GlcNAc was used to coat tissue-culture dishes. Poly[*N*-p-vinylbenzyl-*O*-*α*-d-glucopyranosyl-(1 → 4)-d-gluconamide] (PV-MA), a glucose-bearing polymer, was provided by Celagix Research Ltd. (Yokohama, Japan) [26]. Recombinant human vimentin was produced as a His-tagged recombinant protein according to previously described methods [29].

### 2.2. Evaluation of AC-GlcNAc-Coated Dishes

After the addition of AC-GlcNAc to 96-well plates, 100 *μ*L of 0.002% bovine serum albumin (BSA) was added to each well for blocking for 1 h at 25°C. After blocking, wells were incubated with 100 *μ*L of 0.25 *μ*g/mL biotinylated-succinylated wheat germ agglutinin (sWGA) (Vector Laboratories, Inc., Burlingame, CA, USA) in PBS for 2 h at 25°C. After incubation, the wells were incubated with 100 *μ*L of 0.05 *μ*g/mL horseradish peroxidase- (HRP-) conjugated NeutrAvidin (Thermo Fisher Scientific Inc., Waltham, MA, USA) for 2 h at 25°C, and 100 *μ*L of substrate TMB solution (SeraCare Life Sciences, Inc., Milford, MA, USA) was added to each well. The reaction was stopped by the addition of 100 *μ*L of 1 M HCl solution to each well for 10 min. The absorbance of each well at 450 nm was measured using a microplate reader (iMark, Bio-Rad Laboratories, Inc., Hercules, CA, USA).

### 2.3. Cell Adhesion Assay

UE7T-13 human immortalized MSCs were purchased from the JCRB Cell Bank (Osaka, Japan). AC-GlcNAc-coated wells were blocked with 0.002% BSA for 1 h at 37°C. After blocking, 2 × 10^4^ cells/well in serum-free DMEM were seeded in each well of a 96-well plate and incubated at 37°C with 5% CO_2_ for 1 h. After incubation, nonadherent cells were removed by washing with PBS, and 100 *μ*L of 10% methylthiazole tetrazolium (MTT) assay reagent (Dojindo Laboratories, Kumamoto, Japan)/DMEM containing 10% fetal bovine serum (FBS) was added to each well, followed by incubation for 2 h at 37°C with 5% CO_2_. After 2 h of incubation, MTT formazan was solubilized with 100 *μ*L of dimethyl sulfoxide. The absorbance at 570 nm of these solutions was measured using a microplate reader. The number of adherent cells was determined by the standard curve based on formazan production.

### 2.4. Western Blotting

Cells were lysed in 1% Triton-X100, 20 mM HEPES-NaOH (pH 7.5), 500 mM NaCl, and protease/phosphatase inhibitor cocktail (Nacalai Tesque Inc., Kyoto, Japan), and the soluble fractions were collected by centrifugation at 20000 × *g* for 15 min at 4°C. Fractions were analyzed by SDS-PAGE, and proteins in the gel were transferred to an Immobilon-P PVDF membrane (EMD Millipore, Billerica, MA, USA). Proteins were detected with a mouse monoclonal antivimentin antibody (V9, Sigma) and anti-*β*-actin antibody (Sigma). Detection was performed using a C-DiGit® Blot Scanner (LI-COR Inc., Lincoln, NE, USA). Blotting data were processed using Image Studio Lite software (LI-COR).

### 2.5. Western Blotting of Cell-Surface Vimentin in UE7T-13 Cells

To detect cell-surface expression of vimentin in UE7T-13 cells, biotinylation of total cell-surface proteins was performed by incubation with 500 *μ*g/mL Biotin-AC_5_-Sulfo-Osu (Dojindo) for 20 min at 4°C as described previously [26–29]. After the incubation, total biotinylated proteins were collected using Manosphere™ MS300/Streptavidin (TAKARA BIO Inc., Shiga, Japan). Proteins and whole-cell lysates were analyzed by Western blotting.

### 2.6. Vimentin Knockdown in UE7T-13 Cells

Vimentin-knockdown UE7T-13 cells were produced by transfection with pcDNA 6.2-GW/EmGFP-miR-human vimentin (vimentin-miRNA vectors; Thermo Fisher Scientific Inc.), as previously described [24]. The miRNA sequence was 5′-CACACTTTCATATTGCTGACG-3′ (nucleotides 1230–1250 of human vimentin mRNA, GenBank Accession No. NM_003380). The pcDNA 6.2-GW/EmGFP-miR-neg control plasmid (negative-miRNA vector; Thermo Fisher Scientific Inc.) was used as a negative control. Cells were transfected with each vector using the Neon® Transfection System (Thermo Fisher Scientific Inc.) [29].

### 2.7. Isolation of Bone Marrow Cells

The isolation of rat bone marrow cells from male and female 6–9-week-old Wistar rats was approved by the Institutional Animal Care and Use Committee of University of Tokyo (approval ID: 28-9). The protocol was performed in accordance with all relevant guidelines and regulations. Rats were sacrificed under isoflurane anesthesia, and thigh and shin bones were excised. Bone marrow was isolated by flushing the bones with serum-free medium. Red blood cells in bone marrow were hemolyzed with 0.15 M ammonium chloride, 17 mM Tris-HCl, and 0.25 mM EDTA2Na, pH 7.65. After hemolysis, mononuclear cells were collected by centrifugation at 200 × *g* for 3 min.

### 2.8. Selection of MSCs on AC-GlcNAc-Coated Tissue-Culture Dishes

AC-GlcNAc- and PV-MA-coated dishes were prepared by drying 1 mL of 100 *μ*g/mL AC-GlcNAc in 35-mm tissue-culture dishes. Rat bone marrow cells (mononuclear cell fraction; 3–7 × 10^6^ cells/35-mm dish) were seeded on AC-GlcNAc-coated dishes, PV-MA-coated dishes, and tissue-culture dishes and were cultured with 2 mL of MesenCult Proliferation medium with MesenPure (Mouse) (Veritas). After 7 days of culture, the medium was changed to remove nonadherent cells. Thereafter, the medium was changed every 3–4 days. After 10–17 days, colonies were observed by crystal violet staining, and stained areas were quantitated using ImageJ 1.64r.

### 2.9. Flow Cytometry

Rat bone marrow cells were suspended in 10% FBS/DMEM and incubated with a cell-surface vimentin monoclonal antibody (clone 84-1; 1 : 100; Abnova Corporation, Taipei, Taiwan) at 4°C for 1 h. After incubation, cells were incubated with CF488-conjugated antimouse IgG secondary antibody (1 : 500; Biotium, Hayward, CA, USA) at 4°C for 1 h.

Proliferating cells were harvested by detaching with Accutase (Nacalai Tesque Inc., Kyoto, Japan). Cells were suspended in 10% FBS/DMEM and incubated with FITC-conjugated antirat CD11b/c antibody (1 : 100), FITC-conjugated antirat CD90 antibody (1 : 100), FITC-conjugated antirat CD29 antibody (1 : 100), FITC-conjugated antirat CD44 antibody (1 : 100), PE-conjugated antirat CD54 antibody (1 : 100), or PE-conjugated antirat CD106 antibody (1 : 100); all were purchased from BioLegend, Inc., San Diego, USA. For CD34 and CD45 staining, cells in 10% FBS/DMEM were incubated with anti-CD34 antibody (1 : 100; Santa Cruz Biotechnology Inc., Dallas, TX, USA) or anti-CD45 antibody (1 : 100; Abcam plc., Cambridge, UK) at 4°C for 1 h and then incubated with CF488-conjugated antirabbit IgG secondary antibody (1 : 500; Biotium) at 4°C for 1 h. For CD73 and CD105 staining, harvested cells were fixed with 4% paraformaldehyde (PFA)/PBS. After fixation, the cells were incubated with anti-CD73 (1 : 50; Proteintech Group, Inc., Rosemont, IL, USA) or anti-CD105 (1 : 50; Bioss Antibodies Inc., Woburn, MA, USA) at 4°C for 1 h and then incubated with CF488-conjugated antirabbit IgG secondary antibody (1 : 500; Biotium) at 4°C for 1 h. Mouse IgG (Wako Pure Chemical Industries, Ltd., Osaka, Japan) and rabbit IgG (Thermo Fisher Scientific Inc.) were used as isotype controls. To assess for cellular senescence of MSCs, the harvested cells were cultured with 10% FBS/DMEM for 1 day and stained using the Cellular Senescence Detection Kit-SPiDER-*β*Gal (Dojindo). Stained cells were then analyzed using a Guava EasyCyte Flow Cytometry System (EMD Millipore).

### 2.10. Analysis of MSC Differentiation

Colonies were dissociated using Accutase (Nacalai Tesque) and reseeded on 35-mm tissue-culture dishes. Cells were cultured to 70–80% confluence with MesenCult Proliferation medium. To induce differentiation into adipocytes and osteoblasts, cells were cultured using the MesenCult Adipogenic Differentiation Kit (Mouse) or MesenCult Osteogenic Stimulatory Kit (Mouse) (Veritas) for 3 weeks. Cells were then fixed with 4% PFA/PBS. Alizarin Red (40 mM; Sigma) and Oil Red O (0.5%; Wako) staining was performed. To induce differentiation into chondrocytes, cells were cultured in round-bottom 96-well plates at 1 × 10^4^ cells/well, and cell aggregates were allowed to form for 2 days. After 2 days, these aggregates were cultured with Chondrogenic Differentiation Medium (R&D Systems, Inc., Minneapolis, MN, USA) for 3 weeks. Aggregates were then fixed with 30% ethanol and 2% sucrose/PBS at 4°C overnight and then incubated with 60% ethanol, 40% acetic acid, and 0.1% Alcian Blue (Wako) for 2 h. After incubation, these aggregates were washed with 55% ethanol and 35% acetic acid solution three times.

### 2.11. Statistical Analyses

Data are expressed as means ± standard deviations (SDs) from three or more independent experiments. Differences between two groups were evaluated by unpaired Student's *t*-tests using Microsoft Excel for Mac (version 16.15). Values of *P* < 0.01 were considered significant.

## 3. Results

### 3.1. Optimization of AC-GlcNAc-Coated Polystyrene Dishes

To determine the optimal conditions for AC-GlcNAc coating, we produced AC-GlcNAc of different sizes using RAFT reagents. Seven types of AC-GlcNAc were produced with average molecular weights (*M*_w_'s) of 2600–10300 Da and 8–48 GlcNAc ligands (Figure 1(a) and Table 1). AC-GlcNAc coatings were assessed by the interaction of sWGA, a specific GlcNAc-binding lectin (Figure 1(b)). AC-GlcNAc is easily adsorbed on polystyrene dishes because AC-GlcNAc polymerized by RAFT has an alkyl chain (C_12_H_25_) at the terminal region. The shortest AC-GlcNAc 5- (*M*_w_ 2600-) coated wells had the strongest interactions with sWGA. These results showed that short (low-molecular-weight) AC-GlcNAc 5 and 10 were more stably immobilized on the dishes than were long (high-molecular-weight) AC-GlcNAc 15–50 (Figure 1(b)). It is possible that the effect of the alkyl chain at the terminal region of AC-GlcNAc is greater for short than for long AC-GlcNAc coatings. Based on these results, the optimal polymers for our purposes were AC-GlcNAc 5 (*M*_w_ 2600) and AC-GlcNAc 10 (*M*_w_ 3500).

### 3.2. Expression of Cell-Surface Vimentin and Adhesion of UE7T-13 Cells to AC-GlcNAc-Coated Dishes

To determine whether MSCs adhere to AC-GlcNAc-coated dishes via cell-surface vimentin, the expression of cell-surface vimentin on human immortalized UE7T-13 MSCs was examined by the biotinylation of total cell-surface protein and Western blotting. In the fraction of biotinylated proteins from MSCs pulled down with streptavidin beads, a vimentin band was detected, while *β*-actin was not (Figure 1(c)), as predicted if vimentin is a cell-surface protein. Next, we determined whether UE7T-13 cells adhere to AC-GlcNAc-coated dishes. During incubation at 37°C, 80–90% of the seeded UE7T-13 cells adhered to AC-GlcNAc 5- and 10-coated dishes, which was greater than the number of adherent cells on AC-GlcNAc 15-, 20-, and 30-coated dishes (Figure 1(d)). The adhesion at 4°C was less than that at 37°C; however, adhesion to AC-GlcNAc 5- and 10-coated dishes was greater than adherence with longer polymers (Figure 1(d)).

**Figure 2:**
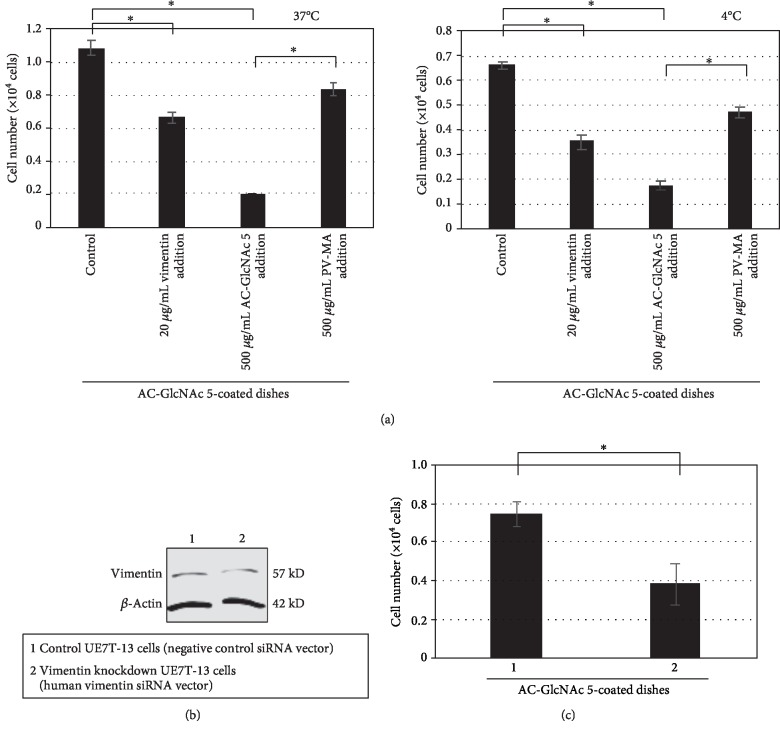
The interaction between cell-surface vimentin and AC-GlcNAc is responsible for the adhesion of UE7T-13 cells to coated dishes. (a) Inhibition of adhesion by AC-GlcNAc at 37 (left) and 4°C (right), *n* = 4. (b) Expression of vimentin in knockdown and control cells. (c) Inhibition of adhesion by vimentin knockdown. Adherence of vimentin-knockdown and control cells to AC-GlcNAc 5-coated dishes at 4°C, *n* = 5. ^∗^*P* < 0.01.

Next, we determined whether the adhesion of UE7T-13 cells to AC-GlcNAc-coated dishes was attributable to the interaction between cell-surface vimentin and GlcNAc. The inhibition of UE7T-13 cell adhesion to AC-GlcNAc 5-coated dishes was examined after the addition of recombinant vimentin, AC-GlcNAc 5, and poly[*N*-p-vinylbenzyl-*O*-*α*-d-glucopyranosyl-(1 → 4)-d-gluconamide] (PV-MA), a glucose-bearing polymer, to the cell suspension. PV-MA is composed of polystyrene as the main chain and glucose as a side chain and does not interact with vimentin [25]. UE7T-13 cells were incubated with 20 *μ*g/mL recombinant vimentin, 500 *μ*g/mL AC-GlcNAc 5, or 500 *μ*g/mL PV-MA for 30 min at 37°C. After incubation, these cell suspensions were incubated on AC-GlcNAc 5-coated dishes for 1 h at 37 or 4°C. Adhesion was estimated by MTT assays. During incubation at 37 and 4°C, adhesion was decreased by approximately 40% by recombinant vimentin and decreased by approximately 80% by AC-GlcNAc (Figure 2(a)). However, the reduction in adhesion was markedly lower with PV-MA than with AC-GlcNAc (Figure 2(a)).

To determine whether vimentin was required for adhesion, vimentin-knockdown and negative control UE7T-13 cells were prepared. After 3 days, vimentin expression on UE7T-13 cells transfected with human vimentin siRNA was significantly lower than control expression (Figure 2(b)). We previously reported that the adhesion of vimentin-knockdown HeLa cells to tissue-culture dishes was decreased at 37°C [28]. Vimentin intracellularly supports the cell-surface expression of some integrins, and the cell-surface expression of integrins was reduced by vimentin-knockdown. To precisely clarify the adhesion of vimentin-knockdown UE7T-13 cells to AC-GlcNAc 5-coated dishes, vimentin-knockdown and negative control UE7T-13 cells (2 × 10^4^ cells) were incubated on AC-GlcNAc 5-coated dishes for 1 h at 4°C. Since the binding of cell-surface vimentin to AC-GlcNAc even occurs at 4°C, the specific adhesion of these cells to AC-GlcNAc 5-coated dishes can be estimated except for integrin interactions. The adhesion of vimentin-knockdown UE7T-13 cells was approximately half that of negative control UE7T-13 cells (Figure 2(c)).

### 3.3. Colony Formation by Bone Marrow Cells on AC-GlcNAc-Coated Dishes

**Figure 3:**
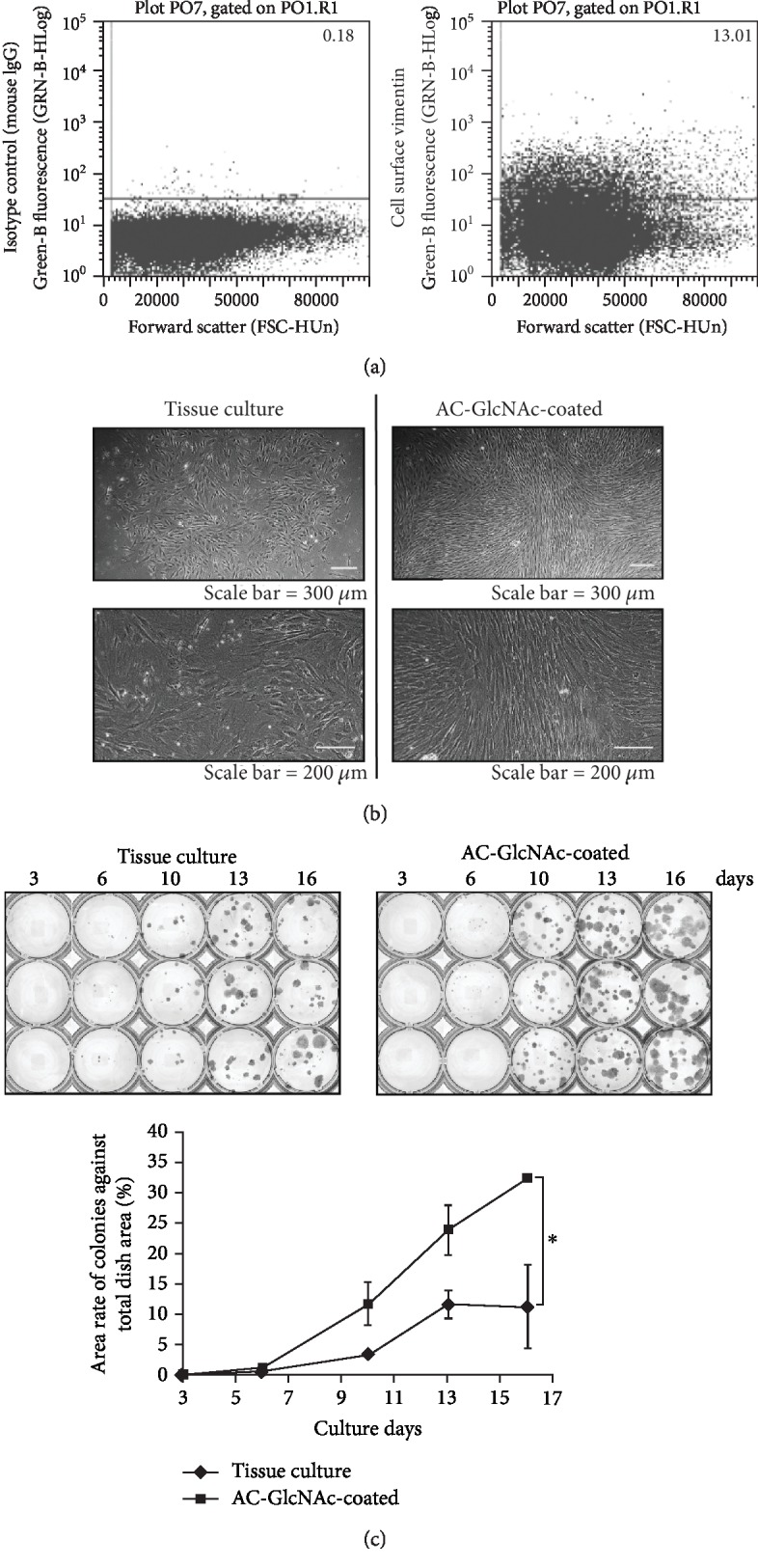
Characteristics of rat bone marrow cells grown on AC-GlcNAc-coated and uncoated tissue-culture dishes. (a) Flow cytometric analysis of expression of cell-surface vimentin in rat bone marrow cells. (b) Phase-contrast images of colonies formed on tissue-culture dishes and AC-GlcNAc-coated dishes at 10 days. Lower images are enlargements of upper images. (c) Images of crystal violet-stained colonies and areas of colonies over 3 to 16 days of culture. ^∗^*P* < 0.01, *n* = 3.

**Figure 4:**
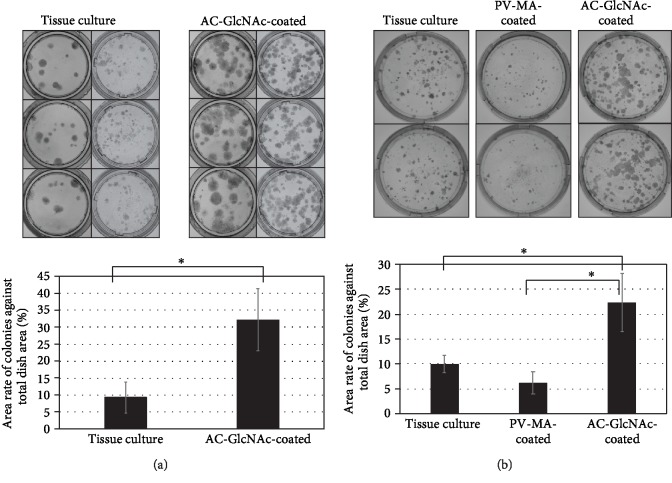
Colony formation of rat bone marrow cells on AC-GlcNAc-coated dishes and tissue-culture dishes. (a) Representative images and areas of colonies after 17 days of culture. ^∗^*P* < 0.01, *n* = 18. (b) Representative images and areas of colonies on AC-GlcNAc-coated dishes, PV-MA-coated dishes, and tissue-culture dishes for 10 days. ^∗^*P* < 0.01, *n* = 3.

First, we analyzed the existence of cell-surface vimentin-expressing cells among bone marrow cells by flow cytometry. Cell-surface vimentin-expressing cells were found at a frequency of 14 ± 2% (*n* = 7) (Figure 3(a)). MSCs are expected to be contained within this stromal-cell population. Next, to determine whether the establishment of MSCs is promoted by specific interactions between MSCs and AC-GlcNAc-coated dishes via cell-surface vimentin, we prepared dishes with 100 *μ*g/mL of AC-GlcNAc 10 solution and 100 *μ*g/mL of PV-MA solution, respectively, because the yield of AC-GlcNAc 10 was better than that of AC-GlcNAc 5 during polymerization. Rat bone marrow cells were cultured on AC-GlcNAc-coated dishes or control tissue-culture dishes.  Figure 3(b) shows the phase-contrast images of colonies formed at 10 days. Cell–cell contacts in the colonies formed on AC-GlcNAc-coated dishes were tight, whereas those on tissue-culture dishes were loose. At 3, 6, 10, 13, and 16 days, colony formation on each dish was investigated by crystal violet staining. At 10 days, more colonies were detected on AC-GlcNAc-coated dishes than on control tissue-culture dishes, and at 16 days, the coated dishes had larger colonies (Figure 3(c)). The proliferation rate decreased on tissue-culture dishes by 13–16 days, while it was maintained in cells on AC-GlcNAc-coated dishes up to 16 days. Finally, the stained areas of colonies on AC-GlcNAc-coated dishes were threefold to fourfold greater than those on control dishes at 17 days (Figure 4(a)).

We speculated that many highly proliferative cells adhered to the coated dishes. Next, we examined whether the adhesion of these proliferative cells was related to their interactions with GlcNAc. Rat bone marrow cells were cultured on PV-MA-coated dishes for 10 days. Many colonies formed on AC-GlcNAc-coated dishes, whereas few colonies formed on PV-MA-coated dishes (Figure 4(b)).

### 3.4. Proliferating Cells on AC-GlcNAc-Coated Dishes Express MSC-Specific Markers

**Figure 5:**
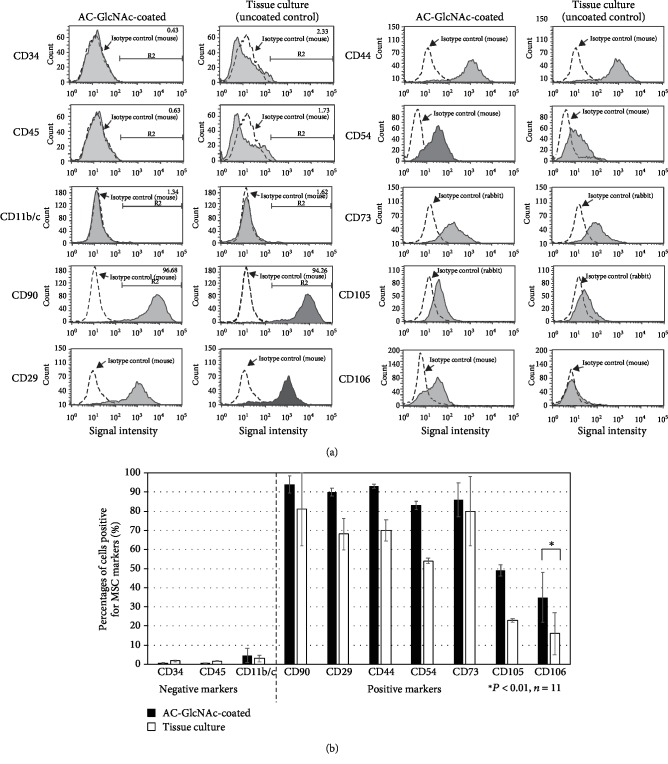
Flow cytometric analysis of proliferating cells from AC-GlcNAc-coated and control uncoated dishes. (a) Representative charts of flow cytometric analysis for expression of MSC markers (CD34, CD45, CD11b/c, CD90, CD29, CD44, CD54, CD73, CD105, and CD106) in the proliferating cells on each dish. (b) Percentages of cells positive for MSC-positive and MSC-negative markers.

To determine whether the colonies that formed on both dishes had the characteristics of MSCs, we examined the expression of seven MSC-positive markers and one MSC-negative marker by flow cytometry. After about 3 weeks of culture of bone marrow cells on AC-GlcNAc-coated and tissue-culture dishes, these proliferating cells were recovered. The passage numbers of these cells were 0 or 1 in all experiments. The proliferating cells from colonies on AC-GlcNAc-coated dishes and control tissue-culture dishes expressed the MSC markers CD90, CD29, CD44, CD54, CD73, and CD105, but not the MSC-negative CD34, CD45, and CD11b/c (Figure 5). CD90-positive cells comprised 94 ± 5% and 81 ± 19%, CD34-positive cells comprised 0.65 ± 0.23% and 1.8 ± 0.46%, CD45-positive cells comprised 0.71 ± 0.09% and 1.6 ± 0.15%, and CD11b/c-positive cells comprised 4.6 ± 3.7% and 3.1 ± 1.7% of the populations from AC-GlcNAc-coated and control uncoated dishes, respectively. The percentages of CD90-, CD29-, CD44-, CD54-, and CD73-positive cells from AC-GlcNAc-coated dishes were all approximately 80%, more than those from control dishes (Figure 5(b)). The percentage of CD105-positive cells from both dishes was lower than that of human MSCs. Since there are no sensitive antirat CD105 antibodies for flow cytometry, we could not observe a high percentage of CD105-positive cells on both dishes. CD106-positive cells from AC-GlcNAc-coated dishes were 35 ± 13% of total cells, while those on control uncoated dishes were 16 ± 11%. Interestingly, the CD106-expression level on AC-GlcNAc-coated dishes was significantly higher than that on control dishes (Figure 5(b)). It has been reported that CD106 is a reliable marker for MSCs because it is not expressed on fibroblasts and because CD106-positive MSCs have high proliferative activity [30, 31]. These results demonstrated that the proliferative cells from AC-GlcNAc-coated dishes had a higher proportion of cells with MSC characteristics than those from control uncoated dishes.

Next, we evaluated multiple concentrations of AC-GlcNAc for coating tissue-culture dishes and isolation of MSCs. Bone marrow cells were cultured on 0, 0.01, 0.1, 1, 10, 100, and 1000 *μ*g/mL AC-GlcNAc-coated dishes for 13 days, and then expression of CD11b/c and CD90 in proliferating cells was examined. The numbers of proliferating cells from 0.01–1000 *μ*g/mL AC-GlcNAc-coated dishes were approximately twofold greater than those from control dishes ([Supplementary-material supplementary-material-1]). The percentages of CD11b/c-positive cells from 10 and 100 *μ*g/mL AC-GlcNAc-coated dishes were appropriately low, approximately 5% ([Supplementary-material supplementary-material-1]). The proportions of CD90-positive cells from 0.01–100 *μ*g/mL AC-GlcNAc-coated dishes were approximately 90%; however, proportions from 1000 *μ*g/mL AC-GlcNAc-coated dishes and tissue-culture dishes were approximately 80% ([Supplementary-material supplementary-material-1]). More contamination of MSCs by other cell types was observed from 1000 *μ*g/mL AC-GlcNAc-coated dishes and control tissue-culture dishes; however, MSCs were isolated with high purity using 10–100 *μ*g/mL AC-GlcNAc. These results suggested that the optimal concentration of AC-GlcNAc was 10–100 *μ*g/mL.

### 3.5. Cellular Senescence in Proliferating Cells Is Reduced by Isolation on AC-GlcNAc-Coated Dishes

**Figure 6:**
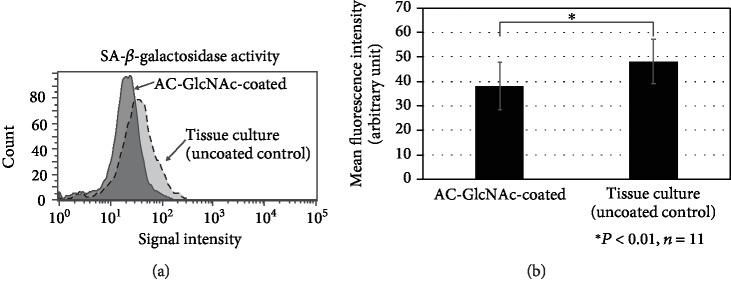
AC-GlcNAc-selected cells have reduced levels of the senescence marker SA-*β*-galactosidase. (a) Representative chart of flow cytometric analysis of SA-*β*-galactosidase activity. (b) SA-*β*-galactosidase activity.

A reduction in the proliferation rate was observed in cells from tissue-culture dishes but not from those on AC-GlcNAc-coated dishes (Figure 3(c)). Therefore, we assessed senescence by measuring senescence-associated *β*-galactosidase (SA-*β*Gal) activity. To perform Cellular Senescence Detection Kit-SPiDER-*β*Gal on these proliferating cells, the proliferating cells after about 3 weeks of culture on AC-GlcNAc-coated and tissue-culture dishes were cultured on tissue-culture dishes for one day. Cells from control uncoated dishes had significantly higher SA-*β*Gal activity than cells from AC-GlcNAc-coated dishes (Figure 6).

### 3.6. Differentiation Efficiency of MSCs Selected by Adherence to AC-GlcNAc

**Figure 7:**
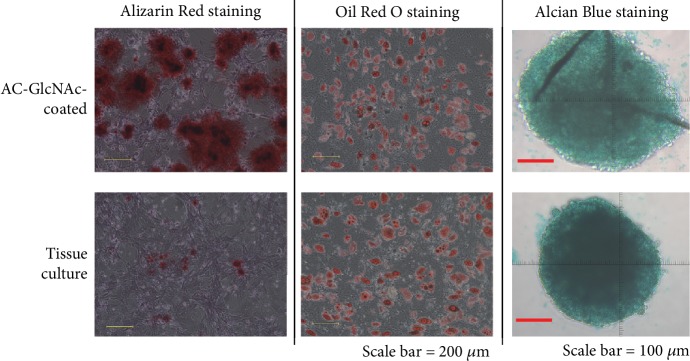
Osteogenic, adipogenic, and chondrogenic differentiation is increased by selection of cells adhering to AC-GlcNAc-coated dishes.

MSCs have the potential for osteogenic, adipogenic, and chondrogenic differentiation media. The ability of MSCs to undergo osteogenic, adipogenic, and chondrogenic differentiation was observed by Alizarin Red, Oil Red O, and Alcian Blue staining of proliferating cells (Figure 7). The proliferating cells recovered from AC-GlcNAc-coated and tissue-culture dishes were cultured on tissue-culture dishes for 3 weeks with osteogenic, adipogenic, and chondrogenic differentiation medium respectively. The large changes in long-term culture of AC-GlcNAc-isolated MSCs were not observed. These data demonstrate that MSCs obtained by AC-GlcNAc-coated dishes had their capacity for differentiation, similar to those by tissue-culture dishes.

## 4. Discussion

We have demonstrated an improved method of isolation of MSCs from bone marrow cells with AC-GlcNAc-coated dishes. Low-molecular-weight AC-GlcNAc 5 and AC-GlcNAc 10 were more easily adsorbed than were longer polymers and facilitated the substantial adhesion of UE7T-13 cells. We speculate that the alkyl chain at the end of AC-GlcNAc, a hydrophobic moiety, is important in binding to polystyrene because the main, hydrophilic chain of AC-GlcNAc 5 and 10 is shorter than that of AC-GlcNAc 15–50. It has previously been reported that AC-GlcNAc, which has approximately 10 GlcNAc units, strongly interacts with vimentin-expressing HeLa cells and has high affinity for vimentin (*K*_D_: 3 × 10^−8^ M) [29]. Therefore, we chose AC-GlcNAc 5- and 10-coated dishes as optimal for MSC isolation.

Many studies have examined the surface expression of vimentin in cell types including fibroblasts, endothelial cells, malignant tumor cells, and circulating tumor cells (CTCs) [20–25, 32–35]. We detected vimentin expression on MSCs. We have previously reported that cell-surface vimentin has GlcNAc-binding activity and that its expression is enhanced by interactions with GlcNAc [28]. Consistent with this hypothesis, it has been suggested that when GlcNAc molecules interact with cell-surface vimentin, the formation of tetramers from vimentin filaments is promoted by the phosphorylation of vimentin, after which the tetramers are recruited to the cell surface [28]. Accordingly, the surface expression of vimentin in our MSCs might be enhanced by our protocol. However, the physiological significance and roles of cell-surface vimentin remain unclear. We have previously proposed the biological function that cell-surface vimentin is involved in engulfment and clearance of dying cells [27]. Cell-surface vimentin was found to interact with *O*-GlcNAc-modified proteins, and *O*-GlcNAc-modified proteins that abundantly exist in the cytoplasm are leaked from dying cells (necrosis and secondary apoptosis). It is considered that cell-surface vimentin engulfs and clears dying cells by binding to *O*-GlcNAc-modified proteins leaked from dying cells. It is reported that endogenous MSCs are recruited to various inflammatory and lesion sites by multiple chemoattractants [36]. We speculate that cell-surface vimentin of MSCs may be involved in the recruitment to these sites by recognizing *O*-GlcNAc-modified proteins released from these sites. Moreover, we previously reported some drug delivery systems based on the interaction of cell-surface vimentin with GlcNAc-conjugated materials [37, 38]. In these previous studies, tumorigenesis and abnormalities induced by the interaction of GlcNAc-conjugated materials with cells were not observed. Therefore, we consider that there may be no risk such as tumorigenesis for the interaction.

MSCs are conventionally isolated from bone marrow cells by adhesion to tissue-culture dishes, with efficiency depending on nonspecific interactions [15, 16, 39]. Many MSC enrichment approaches have been developed, based on expression of NGF receptor [40, 41], STRO-1 antibody-bound antigen [42–44], CD90, and CD73 [17, 18, 45]. Moreover, since MSCs express *β*1-integrin, the enrichment of adherent cells has been performed using fibronectin-coated dishes [39]. Despite the utility of these strategies, large quantities of costly antibodies are needed. Since fibronectin is included in medium containing serum, the use of fibronectin-coated dishes might not be optimal as an enrichment strategy. AC-GlcNAc, on the other hand, is stable and inexpensive, due to its easy synthesis.

Multiple assays suggest that our approach offers a significant improvement over adherence to uncoated dishes. We detected more and larger colonies on AC-GlcNAc-coated dishes than on control dishes. In proliferating cells on both AC-GlcNAc-coated and tissue-culture dishes, osteogenic, adipogenic, and chondrogenic differentiation was robust. Therefore, we consider that AC-GlcNAc-isolated cells stay vigorous. However, the changes in long-term passaging after GlcNAc isolation remain undetermined. Since Figure 6 showed that the expression of SA-*β*-galactosidase in AC-GlcNAc-isolated MSCs was low, we consider that there is no change of AC-GlcNAc-isolated MSCs in long-term passaging.

The expression of MSC markers was higher from AC-GlcNAc-coated dishes, particularly for CD106. CD106-positive MSCs have been reported to possess high cytokine-secretion activity and are involved in immunoregulatory processes including interleukin production and immunomodulatory activities [46]. CD106 in MSCs is a useful surface marker for discriminating MSCs from fibroblasts because many MSC markers, such as CD90, CD73, and CD105, but not CD106, are also expressed on fibroblasts [30, 31, 46]. The expression of CD106 in MSCs is regulated by cell–cell adhesion though N-cadherin [31]. We observed that cell–cell adhesions in colonies on AC-GlcNAc-coated dishes were tighter than those on control uncoated dishes. High expression of CD106 in MSCs on AC-GlcNAc-coated dishes might thus be induced by N-cadherin-dependent adhesion. Finally, SA-*β* galactosidase activity in the proliferating cells on AC-GlcNAc-coated dishes was significantly lower than that on tissue-culture dishes.

These results demonstrate that MSCs with high proliferative potential are more efficiently isolated using AC-GlcNAc-coated dishes and that vimentin expression on the surfaces of bone marrow cells is a marker for high MSC proliferative activity. AC-GlcNAc coatings on tissue-culture dishes might be unstable, because polystyrene is slightly hydrophilic and the hydrophobic interaction between AC-GlcNAc and these dishes is weak. We expected AC-GlcNAc to be eliminated by long-term incubation with culture medium. However, since extracellular matrix (ECM) components such as fibronectin and collagen are needed for cell proliferation on dishes, we speculated that AC-GlcNAc may play its most important role in the initial adhesion of MSC but that ECM deposition might be important for subsequent MSC proliferation, displacing AC-GlcNAc later in the culture period.

To realize the proliferation and specific adhesion of MSCs to AC-GlcNAc-coated dishes, the optimal concentration of AC-GlcNAc was determined to be 10–100 *μ*g/mL. MesenCult Proliferation medium with MesenPure (Mouse) was observed to facilitate the enhanced proliferation of MSCs from rat bone marrow cells. Other proliferation media for MSCs may be useful for the establishment of MSCs using AC-GlcNAc-coated dishes; however, serum-free medium might be preferable for AC-GlcNAc-coated dishes because the interaction between MSCs and AC-GlcNAc is inhibited by albumin in serum.

## 5. Conclusions

Our results suggest that affinity purification of MSCs from bone marrow cells via interactions between AC-GlcNAc and cell-surface vimentin is an advantageous, inexpensive, and convenient approach for the effective isolation of high-quality MSCs. Our approach is likely to be applicable for the isolation of mouse and human MSCs from other tissues, such as peripheral blood and adipose tissue, as well. Accordingly, this approach is a promising development in the realization of regenerative medicine.

## Figures and Tables

**Table 1:** Polymerization of AC-GlcNAc monomer using reversible addition-fragmentation chain transfer (RAFT) reagents.

Entry	Ratio (monomer/RAFT^a^)	*M*_w_^b^	*M*_n_^c^	*M*_w_/*M*_n_	Number of carbohydrates per polymer^d^
AC-GlcNAc 5	5	2600	3100	1.2	8
AC-GlcNAc 10	10	3500	4800	1.4	13
AC-GlcNAc 15	15	4100	6300	1.5	17
AC-GlcNAc 20	20	5000	8100	1.6	22
AC-GlcNAc 30	30	6600	11400	1.7	32
AC-GlcNAc 40	40	8100	16000	1.9	45
AC-GlcNAc 50	50	10300	17300	1.6	48

^a^2-(Dodecylthiocarbonothioylthio)-2-methylpropanoic acid (DTMPA). ^b^Weight-average molecular weight (*M*_w_). ^c^Number-average molecular weight (*M*_n_). ^d^Numbers of carbohydrates per polymer = (*M*_w_–364 [DTMPA])/346 [AC-GlcNAc monomer].

## Data Availability

All the data used to support the findings of this study are available from the corresponding author upon request.

## Supplementary Materials

Supplementary Materials Figure S1: cell number of the proliferated cells in 0–1000 *μ*g/mL AC-GlcNAc-coated dishes at 13 days. Rat bone marrow cells (3 × 10^6^ cells) were seeded on 0–1000 *μ*g/mL AC-GlcNAc-coated dishes and were cultured for 13 days. Figure S2: flow cytometric analysis for CD11b/c expression of the proliferated cells in 0–1000 *μ*g/mL AC-GlcNAc-coated dishes at 13 days. The lower bar graph shows the percentage (%) of CD11b/c-positive cells. Figure S3: flow cytometric analysis for CD90 expression of the proliferated cells in 0–1000 *μ*g/mL AC-GlcNAc-coated dishes at 13 days. The lower bar graph shows the percentage (%) of CD90-positive cells.
