# Supplementary material for: Improved Isolation of Mesenchymal Stem Cells Based on Interactions between N-Acetylglucosamine-Bearing Polymers and Cell-Surface Vimentin
Source: Stem Cells Int. 2019 Nov 11;2019:4341286. doi: 10.1155/2019/4341286 (PMC6878802; doi:10.1155/2019/4341286)
Supplement: Supplementary Materials — Figure S1: cell number of the proliferated cells in 0–1000 μg/mL AC-GlcNAc-coated dishes at 13 days. Rat bone marrow cells (3 × 10^6^ cells) were seeded on 0–1000 μg/mL AC-GlcNAc-coated dishes and were cultured for 13 days. Figure S2: flow cytometric analysis for CD11b/c expression of the proliferated cells in 0–1000 μg/mL AC-GlcNAc-coated dishes at 13 days. The lower bar graph shows the percentage (%) of CD11b/c-positive cells. Figure S3: flow cytometric analysis for CD90 expression of the proliferated cells in 0–1000 μg/mL AC-GlcNAc-coated dishes at 13 days. The lower bar graph shows the percentage (%) of CD90-positive cells. [file 4341286.f1.pdf]

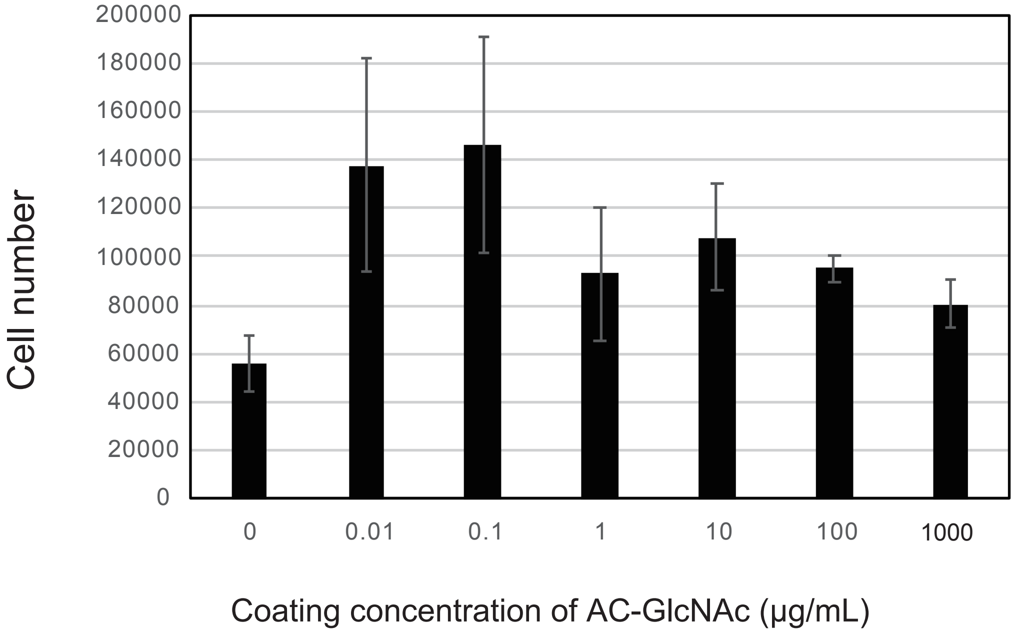

Figure S1: Cell number of the proliferated cells in 0 – 1000 μg/mL AC-GlcNAc-coated dishes at 13 days. Rat bone marrow cells (3×10^6 cells) were seeded on 0 – 1000 μg/mL AC-GlcNAc-coated dishes and were cultured for 13 days.

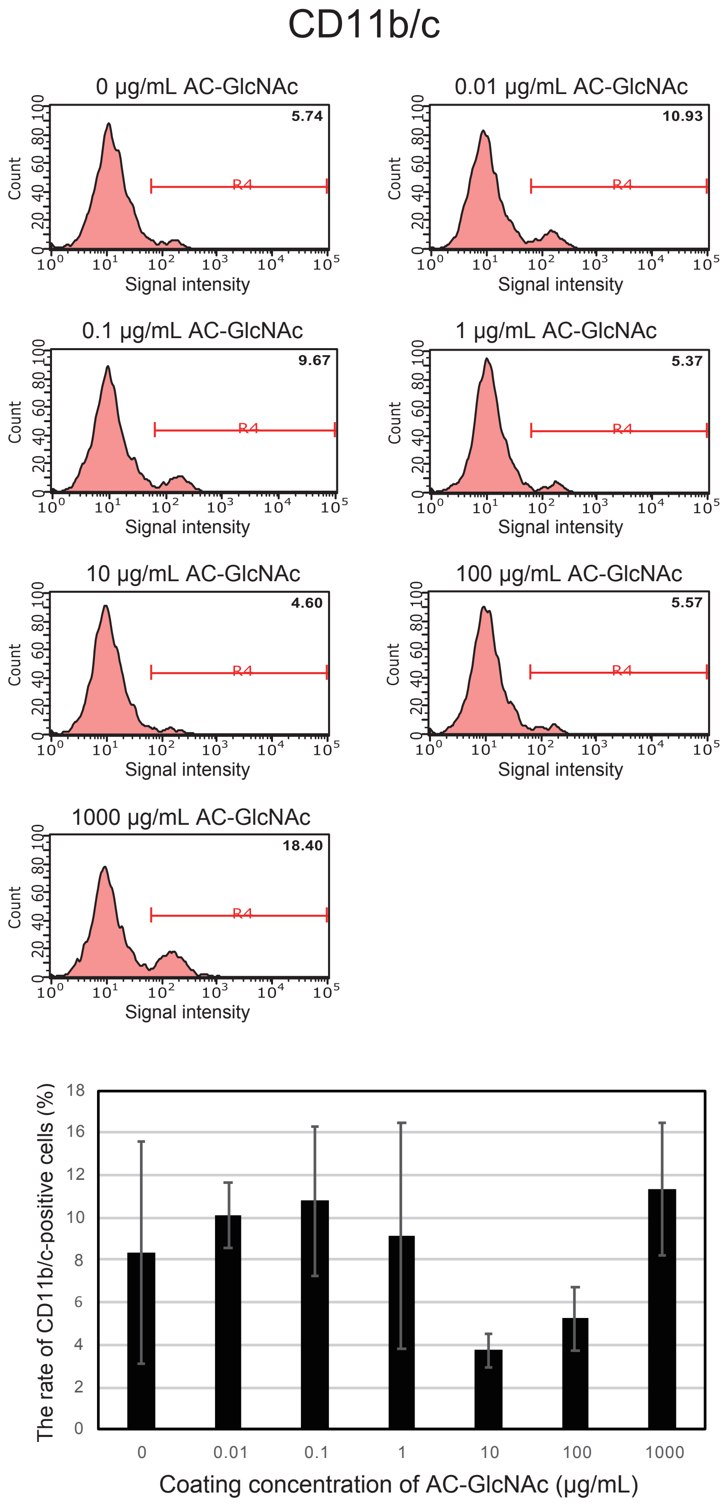

Figure S2: Flow cytometric analysis for CD11b/c expression of the proliferated cells in 0 – 1000 µg/mL AC-GlcNAc-coated dishes at 13 days. The lower bar graph showed the percentage (%) of CD11b/c-positive cells.

CD90

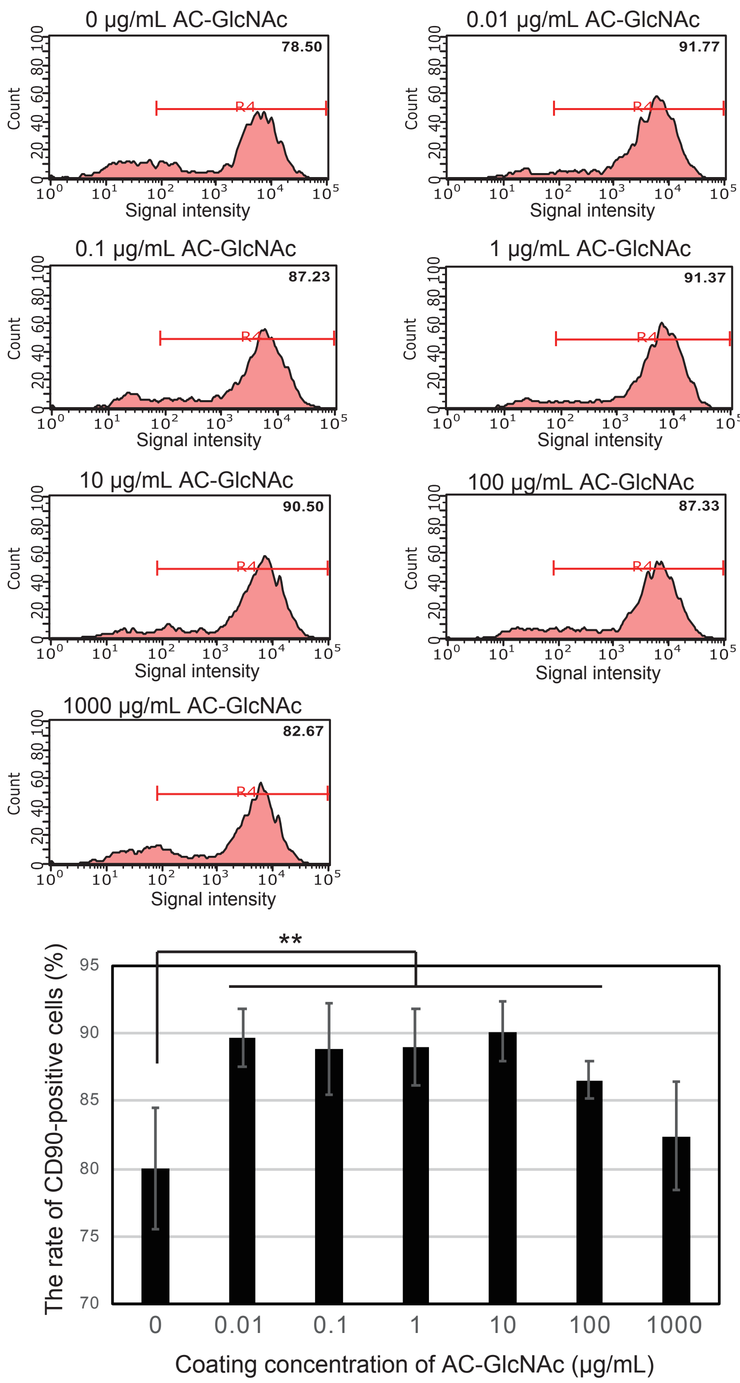

**P<0.05, n = 3

Figure S3: Flow cytometric analysis for CD90 expression of the proliferated cells in 0 – 1000 μg/mL AC-GlcNAc-coated dishes at 13 days. The lower bar graph showed the percentage (%) of CD90-positive cells.
